# Prognostic Value of MicroRNAs in Preoperative Treated Rectal Cancer

**DOI:** 10.3390/ijms17040568

**Published:** 2016-04-15

**Authors:** Azadeh Azizian, Ingo Epping, Frank Kramer, Peter Jo, Markus Bernhardt, Julia Kitz, Gabriela Salinas, Hendrik A. Wolff, Marian Grade, Tim Beißbarth, B. Michael Ghadimi, Jochen Gaedcke

**Affiliations:** 1 Department of General, Visceral, and Pediatric Surgery, University Medical Center Göttingen, Robert-Koch-Str. 40, Göttingen 37075, Germany; azadeh.azizian@med.uni-goettingen.de (A.A.); ingo-epping@web.de (I.E.); jo.peter@chirurgie-goettingen.de (P.J.); markus.bernhardt@med.uni-goettingen.de (M.B.); marian.grade@med.uni-goettingen.de (M.G.); mghadim@uni-goettingen.de (B.M.G.); 2 Department of Medical Statistics, University Medical Center Göttingen, Robert-Koch-Str. 40, Göttingen 37075, Germany; frank.kramer@med.uni-goettingen.de (F.K.); tim.beissbarth@med.uni-goettingen.de (T.B.); 3 Department of Pathology, University Medical Center Göttingen, Robert-Koch-Str. 40, Göttingen 37075, Germany; j.kitz@med.uni-goettingen.de; 4 Department of Developmental Biochemistry, University of Göttingen, Göttingen 37075, Germany; gsalina@gwdg.de; 5 Medical Practice Radiotherapy München, Burgstraße 7, München 80331, Germany; hendrik.wolff@med.uni-goettingen.de

**Keywords:** rectal cancer, prognosis, tumor regression grade, biomarkers, miRNA, chemoradiotherapy

## Abstract

Background: Patients with locally advanced rectal cancer are treated with preoperative chemoradiotherapy followed by surgical resection. Despite similar clinical parameters (uT2-3, uN+) and standard therapy, patients’ prognoses differ widely. A possible prediction of prognosis through microRNAs as biomarkers out of treatment-naïve biopsies would allow individualized therapy options. Methods: Microarray analysis of 45 microdissected preoperative biopsies from patients with rectal cancer was performed to identify potential microRNAs to predict overall survival, disease-free survival, cancer-specific survival, distant-metastasis-free survival, tumor regression grade, or nodal stage. Quantitative real-time polymerase chain reaction (qPCR) was performed on an independent set of 147 rectal cancer patients to validate relevant miRNAs. Results: In the microarray screen, 14 microRNAs were significantly correlated to overall survival. Five microRNAs were included from previous work. Finally, 19 miRNAs were evaluated by qPCR. miR-515-5p, miR-573, miR-579 and miR-802 demonstrated significant correlation with overall survival and cancer-specific survival (*p* < 0.05). miR-573 was also significantly correlated with the tumor regression grade after preoperative chemoradiotherapy. miR-133b showed a significant correlation with distant-metastasis-free survival. miR-146b expression levels showed a significant correlation with nodal stage. Conclusion: Specific microRNAs can be used as biomarkers to predict prognosis of patients with rectal cancer and possibly stratify patients’ therapy if validated in a prospective study.

## 1. Introduction

Colorectal cancer is the third most common cancer worldwide accounting for 1.36 million newly diagnosed cases in 2012 [1]. About 30% of all colorectal cancers are rectal cancers. The treatment of rectal cancer patients depends on the stage of the disease. While patients with Union for International Cancer Control (UICC) stage I and II receive a primary surgery, others with locally advanced rectal cancer are treated first with preoperative chemoradiotherapy (CRT) followed by surgery (total mesorectal excision (TME)). However, within the respective UICC stages, the prognosis of patients may differ. This is partly due to the fact that the UICC staging relies mostly on imaging techniques that are not always able to discriminate appropriately. In addition, patients´ response to the preoperative treatment is heterogeneous. An adequate prediction of response and prognosis in addition to the UICC staging could serve to adapt the therapy modalities individually within the UICC stages and also to individualize the follow-up care to the predicted prognosis. Patients with poor prognosis could receive a more aggressive CRT (e.g., additional oxaliplatin) possibly accepting more severe side effects in order to achieve a better outcome. Furthermore, they could benefit from more frequent follow-up visits to detect early possible disease recurrence as defined by the occurrence of local recurrence or distant metastasis. To predict the prognosis of patients, reliable biomarkers are needed. These can be, for instance, specific DNA mutations, RNA sequences, or proteins. While proteins are subject to many post-transcriptional modifications and DNA sequences do not necessarily reflect the gene expression, some RNA molecules are promising candidates: in contrast to mRNA and tRNA, mature microRNAs (miRNAs) are highly conserved between species [2], very robust towards temperature and pH-values [3]. Due to their apparent proximity to chromosomal breakpoints [4] and their dysregulated expression levels in many malignancies [5], miRNAs were linked to tumorigenesis early after their discovery.

miRNA are small single-stranded non-coding RNAs, about 20–22 nucleotides in length, which play an important role in the post-transcriptional regulation of mRNA [6]. They bind to the targeting mRNA sequence within a complex of specific proteins, called RNA-induced silencing complex (RISC), and induce a cleavage or repression of the target gene [7]. Therefore miRNAs play an important role in the gene expression, and influence many physiological and pathophysiological processes. These qualities make them promising candidates for biomarkers in tumor specimen and liquid biopsies. In 2003, Michael *et al.*, found, for the first time, miRNAs being associated with colorectal cancer [8]. Since then, several studies used miRNAs trying to predict different clinical parameters [9,10,11]. In 2012, we identified a rectal cancer specific miRNA-panel characterizing differences between tumors and normal adjacent mucosa [12]. Eleven of the identified microRNAs were also found by Li *et al.* [13] in the same year. While many following studies investigated a specific miRNA-panel for colorectal cancer and others tried to find miRNAs specific for each UICC stage, a large part of research was investigating miRNAs for predicting response to CRT [14,15,16,17].

Prognosis of patients depends on several known factors: UICC stage, tumor regression grade (TRG), nodal stage, and surgical margins. A large part of these factors, namely TRG, nodal stage, and the quality of the surgical margins, is not known until after CRT and surgery. A that point, the large part of the treatment is already performed and possible side-effects of the preoperative CRT and surgery can not be undone. Therefore, these factors can only have little impact on individualizing the therapy. Our aim is to predict patients’ prognoses in advance, before any treatment. This way, we would be able to stratify the therapy in a way patients would benefit from the most. In the present work, we aimed to explore the impact of miRNAs as biomarkers to predict the patients’ prognosis and response to CRT analyzed in biopsies, which were taken prior to any treatment. All patients were enrolled or treated according to the CAO/ARO/AIO-94 [18,19,20] and CAO/ARO/AIO-04 [21,22] trial of the German Rectal Cancer Study Group.

## 2. Results

### 2.1. Microarray Analysis Identified 14 miRNAs Significantly Associated with Overall Survival

First, we performed microarray analyses of 45 microdissected pretherapeutic biopsies from patients with rectal cancer to identify potential microRNAs with a prognostic value. The clinical data of the patients, including gender, age, UICC stage, cancer-specific survival (CSS), local recurrence (LR), distant-metastasis-free survival (DMS), and disease-free survival (DFS) are summarized in Supplementary Table S1A.

Time-to-event analyses were performed on a gene-by-gene basis, associating the survival times of patients with the expression level of each feature on the microarray chips using Cox Proportional Hazard Ratio. For 14 miRNAs, we could find a significant association to at least two of four survival parameter (overall survival (OS), DFS, CSS or DMS) with *p* < 0.05 (illustrated in Supplementary Figure S1). Those were chosen for further validation as they were considered as promising candidates with probable prognostic or predictive value in rectal cancer patients due to their expression level.

Further, this list of miRNAs was supplemented by five miRNAs (miR-198, miR-223, miR-320a, miR-34b, and miR-497), which showed a possible predictive value in rectal cancer in previous unpublished microarray analysis results of our group and literature research [12,23,24]. In order to further validate our findings, we collected 147 samples and tested the 19 miRNAs using qPCR.

### 2.2. Expression Levels of 19 miRNAs Were Analyzed in 147 Samples: miR-515-5p, miR-573, miR-579, and miR-802 Were Significantly Correlated to Overall Survival and Cancer-Specific Survival

**Figure 1:**
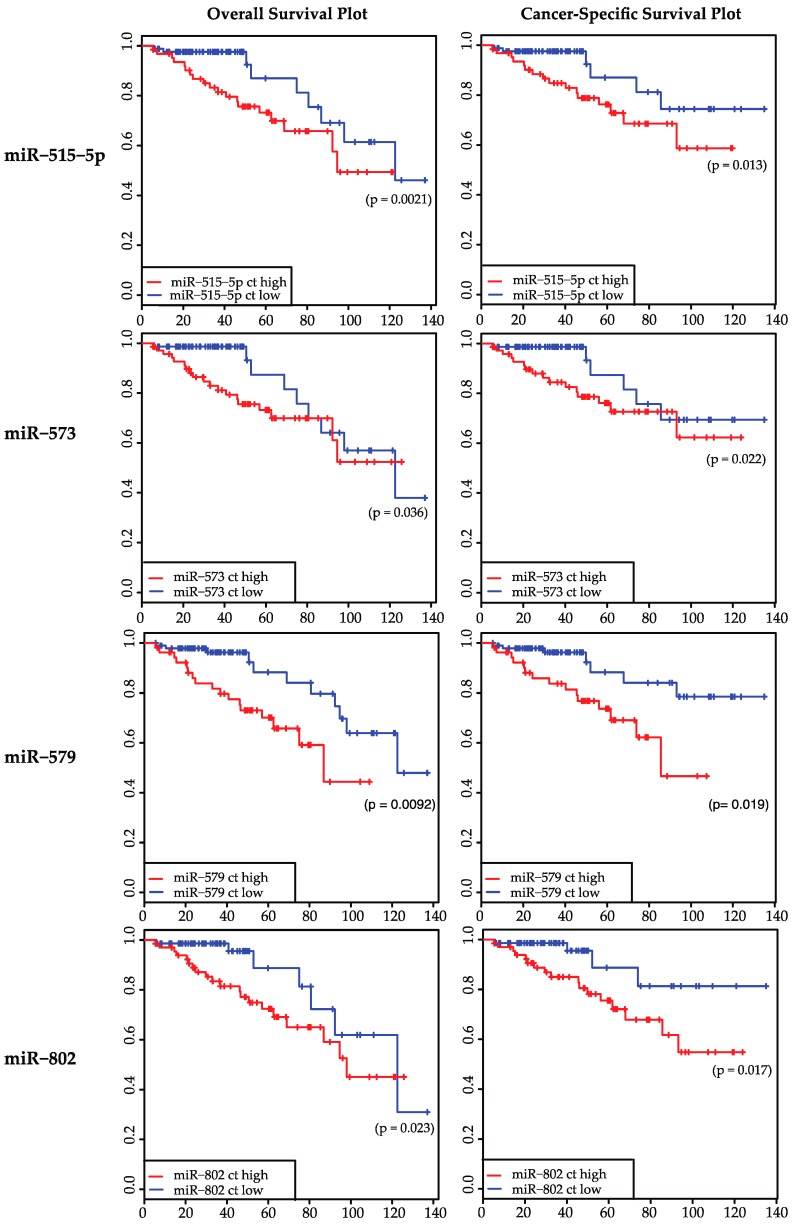
Correlation of miR-515-5p, miR-573, miR-579, and miR-802 to overall survival an cancer-specific survival. Overall survival (**left**) and cancer-specific survival (**right**) of a set of 147 patients are illustrated as Kaplan-Meyer-curves for miR-515-5p, -573, -579, and -802. Red curve represents patients with a high expression of the mentioned miRNAs, blue curve those with a low expression. In all shown cases a high expression of the corresponding miRNA is significantly associated with a worse overall survival and cancer-specific survival.

**Figure 2:**
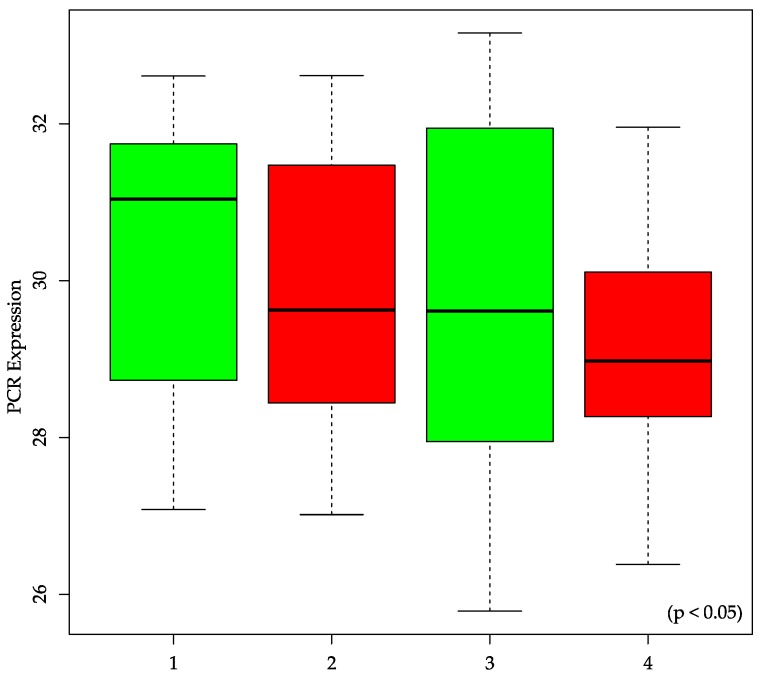
Correlation of miR-573 expression to tumor regression grade (TRG). A high tumor regression grade (TRG 4), which shows a good response to chemoradiotherapy (CRT), is significantly associated to a low expression of miR-573 (*p* < 0.05). Patients with a high expression of miR-573 show a low response to CRT (TRG 1).

The expression levels of the 19 miRNAs (listed in Table 1) in biopsies of rectal cancer tumor tissue (*n* = 147 patients, one sample per patient) were analyzed via qPCR and compared with the clinical parameters OS, CSS, DFS, DMS, and postoperative nodal stage (ypN). Four miRNAs, namely miR-515-5p, miR-573, miR-579 and miR-802, were associated significantly with OS and CSS (*p* < 0.05). Of these four miRNAs, only miR-573 was also associated significantly with the TRG (*p* = 0.0416), which has a known association to the survival of patients [25]. miR-515-5p, miR-573, miR-579, and miR-802 were not able to discriminate between good and poor DFS, DMS, or ypN. All *p*-values of the analyzed miRNAs are summarized in Table 1. Figure 1 shows the survival curves miR-515-5p, miR-573, miR-579, and miR-802 separately. Figure 2 shows the discrimination between TRG 1-4 for miR-573.

### 2.3. miR-133b Is Significantly Associated with Distant-Metastasis-Free Survival

**Figure 3:**
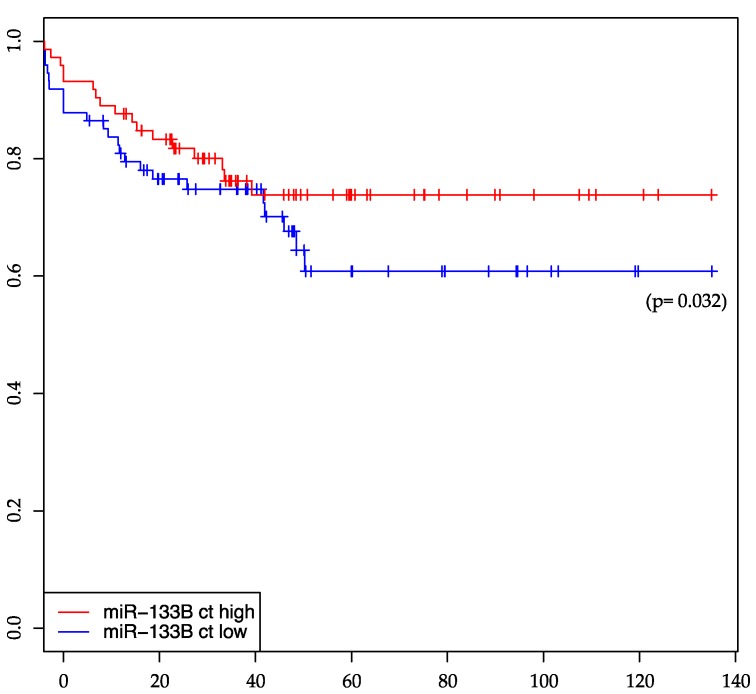
miR-133b distant-metastasis-free survival plot.

For miR-133b expression in treatment-naïve rectal cancer tumor biopsies, a significant association with DMS has been shown (*p* = 0.032). Patients with a low expression level of miR-133b develop more frequently distant-metastasis. miR-133b is the only miRNA (among the investigated miRNAs in this study) being significantly associated to distant-metastasis-free survival, while it does not show any significant association with other clinical parameters according to our analysis. Figure 3 shows distant-metastasis-free survival dependent on a high and low expression of miR-133b in the tumor biopsies, respectively.

### 2.4. miR-146b Expression Levels Show a Significant Association with a Negative Post-Therapeutic Nodal Stage (ypN0)

While miR-146b was not associated with OS, DFS, CSS, DMS, or TRG, it showed a significant association with a negative nodal stage after preoperative CRT and surgery, assessed by the pathologist. However, since we do not know the nodal stage before CRT exactly—this can only be estimated by imaging techniques and radiologists—there is no comparison possible. Therefore, the association between the miR-146b expression and ypN does not make any statement concerning the response to CRT.

## 3. Discussion

Since the treatment of locally advanced rectal cancer includes a preoperative CRT, to which patients respond differently, the aim to find valid biomarkers to predict response in advance has driven many studies. Not only the response towards CRT is heterogeneous but also the prognoses of patients within the UICC stages. Possible biomarkers in blood or cancer biopsies taken prior to any therapy may help to stratify the therapy or adapt the postoperative follow-up examinations in terms of frequency and invasively. Here, miRNAs are one of several possible approaches: Different possible biomarkers were associated with therapy response: fibroblast growth factor receptor 2 (*FGFR2*) [26], β-catenin, vascular endothelial growth factor, and apoptotic protease activating factor 1 [27], expression of *DNAJC12* [28], altered DNA methylation [29], and neutrophil-lymphocyte ratio [30]. Concerning survival prognosis, most studies rely on clinical parameters [31,32,33], but also some molecular parameters were evaluated [34,35,36,37,38].

In the present work, we showed that miR-515-5p, miR-573, miR-579, and miR-802 were correlated significantly with overall survival and cancer-specific survival (*p* < 0.05) of patients with locally advanced rectal cancer treated with a preoperative CRT followed by surgery with quality controlled nerve-sparing TME (according to the study protocol).

These four miRNAs are expressed independently from each other, meaning they do not belong to a common cluster. Although miR-515-5p, located on chromosome 19, belongs to a cluster with 42 members, none of the other three miRNAs (miR-573, miR-579 or miR-802) belongs to that cluster. miR-515 is yet investigated only in few studies and has not been reported in rectal cancer: in 2010, miR-515 was one of 13 miRNAs found overexpressed in oral carcinoma [39]. Follicular tumors showed a distinct overexpression of several members of miR-515 family [40]. These studies together with our work suggest a tumor promoting role of miR-515. But a recent study showed that miR-515-5p inhibits cancer cell migration and metastasis by targeting microtubule affinity regulating kinase 4 (*MARK4*) in breast cancer [41]. A different breast cancer study revealed *IGF-1R* gene as another target for 515-5p [42].

Recently, miR-573 is found to down-regulate the oncogene Tetraspanin 1 (*TSPAN1*) in gastric cancer [43]. Also, in a study concerning breast cancer, miR-573 was found to be down-regulated in *BRCA 1/2*-related breast cancer [44]. Wang *et al.* found that miR-573 targets the melanoma cell adhesion molecule (*MCAM*) and regulates this way melanoma progression [45]. They showed *in vivo* using a murine xenograft model that overexpression groups had lower rates of tumor growth compared with the control group. Taking these studies together, miR-573 seems to perform a tumor suppressive function in diverse cancers. However the results of our work, where we investigated miR-573 for the first time in rectal cancer, show a higher expression level of miR-573 associated with poor overall survival and cancer-specific survival (see Figure 1), suggesting a tumor promoting role. This might show tissue specific effects of miR-573, which can be oppositional in different tumor entities.

The role of miR-579 in cancer cells is poorly investigated yet. Quinn *et al.* concluded in their review about the role of miRNA in regulation of endotoxin tolerance that miR-579 plays a role in regulating the TLR4 signaling pathway during the development of endotoxin tolerance at receptor, signaling pathway, gene transcription and translational levels [46]: miR-579 blocks the translation of TNF-α [47]. According to our results, a high expression of miR-579 in rectal cancer tissue is significantly associated with a poor overall survival and cancer-specific survival (see Figure 1). Further investigations concerning target genes or function in cancer cells needs to be done for miR-579.

Being located on chromosome 21, miR-802 is consequently of great interest in down syndrome associated research [48,49], where it is shown to target the methyl-CpG-binding protein (*MeCP2*), which is underexpressed in down syndrome brains [50]. Also, an association between miR-802 and diabetes was found by Kornfeld *et al.*, who showed that obesity-induced overexpression of miR-802 compromises glucose metabolism through silencing of *Hnf1b* [51]. Nevertheless, it also seems to be of importance in several cancer types: It is supposed to suppress breast cancer proliferation by targeting and suppressing *FoxM1* [52] and showed a moderate tumor suppressive activity in liver cancer cells [53]. On the other hand, Cao *et al.*, claim that miR-802 promotes osteosarcoma cell proliferation by targeting p27 [54]. Another study shows that miRNA‑802 targets the tumor suppressor menin and promotes lung carcinoma proliferation [55]. According to our work, a high expression of miR-802 in rectal cancer tissue is associated significantly with a poor overall survival and cancer-specific survival (Figure 1).

We showed a significant association of miR-133b expression in rectal cancer tumor tissue with a distant-metastasis-free survival of the patients. miR-133b is located on chromosome 6, with the seed sequence TTGGTCC, which is unique and not shared (as far as miRNAs are sequenced till today) between other miRNAs in a so called “seed-family”. In addition, miR-133b does not belong to any bigger cluster of microRNAs (as far as present studies demonstrate), indicating a unique role of this miRNA. miR-133b is declared to have tumor suppressor functions in several studies [56,57,58]. The association of a high expression of miR-133b in rectal cancer with good distant-metastasis-free survival, as shown in our data, is supported by different studies: Qiu *et al.* showed that miR-133b (and to other miRNAs) suppressed the proliferation, migration, invasion and cell cycle progression in gastric cancer cells through decreasing expression of the transcription factor specificity protein 1 (*Sp1*) and its downstream proteins using human gastric cancer cell lines SGC7901, MKN45 and BCG823 [59]. Also, Zhao *et al.* showed for gastric cancer that miR-133b is frequently down-regulated in these tumor biopsies and that its overexpression in gastric cancer cell lines reduces the metastatic potential [60]. They identified the transcriptional factor Gli1 as a direct target of miR-133b: level of Gli1 protein but not mRNA was decreased by miR-133b. In colon cancer cell lines (HT-29 and SW-620) miR-133b was shown be essential for the inhibitory effects of TAp63 on RhoA, E-cadherin and vimentin [61], the authors concluded that miR-133b is able to suppress the metastasis of colon cancer. Also in non-cancer cells, miR-133b seems to correlate inversely to proliferation markers (e.g., Ki67 and cleaved-CK18) suggesting an anti-proliferative role for miR-133b [62]. This data along with our results reveal that miR-133b has an anti-proliferative effect in general, which can be shown in cancer but also in cardiac diseases [63], muscle regeneration [64,65] and embryonic development.

For miR-146b we showed a significant association with the post-CRT nodal stage (ypN) diagnosed by experienced pathologists. A high miR-146b expression is associated with a negative post-CRT nodal stage. According to several studies miR-146b seems to play an important role papillary thyroid carcinoma: Geraldo *et al.* [66] showed a significantly poorer overall survival for patients with higher miR-146b expression levels compared to those with lower miR-146b levels. Using BCPAP, a human papillary thyroid cancer cell line model, they showed that an overexpression of miR-146b significantly increases cell migration and invasiveness, along with increased resistance to chemotherapy-induced apoptosis [66]. Hardin *et al.* [67] analyzed plasma-derived miRNAs in patients with human papillary carcinoma and showed a higher level of miR-146b in patients compared to those with benign lesions. Furthermore, they also showed that the levels of miR-146b increased in proportion to tumor size [67]. Other studies confirmed its role in thyroid cancer [68,69,70,71]. In other cancer types, miR-146b seems to be involved: Li *et al.* reported that miR-146b promotes cell proliferation and inhibits cell apoptosis in esophageal cancer cell lines [72], whereas in glioma miR-146b also seems to perform a tumor suppressor function [73,74].

Other studies have also analyzed the value of miRNAs to predict prognosis or therapy response. miR-21-5p was shown to be overexpressed in complete responders [17]. However, the cohort was rather small (*n* = 43). Another study investigated miR-21 for the same purpose but could not confirm the predictive value of miR-21 [75]. High levels of miR-125b and miR-137 in tumor tissue are shown to be associated with a worse response to CRT [15], although in a small cohort (*n* = 31). Also miR-16, miR-590-5p and miR-153 were associated with therapy response by Kheirelseid *et al.* [76]. Other studies identified miRNA profiles that seem to correlate with response to neoadjuvant chemoradiotherapy [77]. A possible reason for the different identified miRNAs may be the difference between the tissues preservation methods (formalin-fixed paraffin-embedded (FFPE), RNAlater, fresh frozen biopsies) and analyzing methods (qPCR, microarray). Several studies focused on miRNAs and response to targeted anti epidermal growth factor receptor (anti-*EGFR*) therapy, which is a well-established therapy in *KRAS*-wildtype metastatic CRC. However, even in *KRAS*-wildtype patients only approximately 40% respond to this therapy. miR-31 and miR-181 were associated with response to anti-*EGFR* therapy [78,79]; a let-7 microRNA-binding site polymorphism in *KRAS*, the let-7 complementary site 6 (*LCS6*)-variant, also seems to have an impact on response to anti-*EGFR* therapy, though in this regard the study results remain contradictory [80,81].

Taken our results together, we identified six miRNAs with a predictive value concerning patients’ prognosis. High expression levels of miR-515-5p, miR-573, miR-579 and miR-802 showed all a significant correlation to a poor OS and CSS. These four miRNAs could serve together as a miRNA profile to identify patients with a poor outcome, if validated prospectively.

We used two different types of preservation (FFPE for the first set of 45 patients and RNAlater for the validation set with 147 patients), to overcome biases arising from the one or the other type of preservation. Also, we used microarray analysis first and than qPCR to confirm our results. Thus, the identified miRNAs showed their prognostic value in both FFPE and RNAlater, and also in both microarray analysis and qPCR, which strengthens their impact.

However, the identified miRNAs seems to have an impact in other cancer types too, therefore none of them is a rectal cancer specific miRNA. Besides, in some cases, the investigated miRNAs play more of an oppositional role in rectal cancer than in other cancer types. Furthermore, for some of these miRNAs the target genes and functions still need to be investigated.

## 4. Experimental Section

### 4.1. Patients and Samples

**Figure 4:**
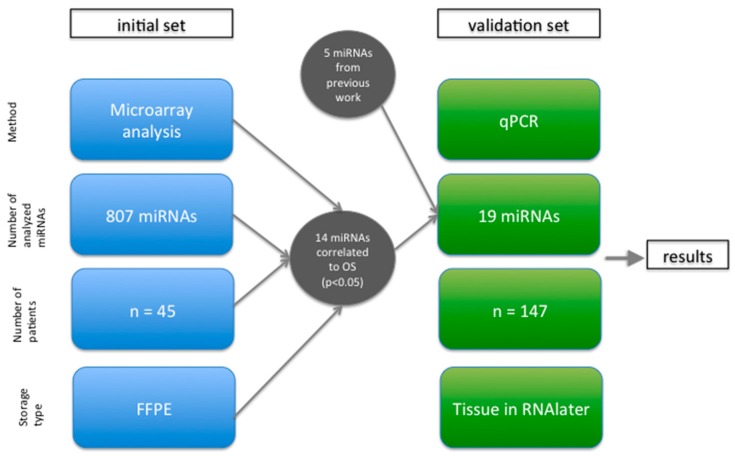
Study design: On the left, the initial set of 45 patients’ formalin-fixed paraffin-embedded (FFPE) samples analyzed via microarray analysis is shown. The results of that analysis showed a significant correlation (*p* < 0.05) of 14 miRNAs to overall survival (OS). These miRNAs together with 5 further miRNAs from previous work were analyzed in 147 patients’ samples (preserved in RNAlater), which were analyzed via quantitative real-time polymerase chain reaction (qPCR). The results are explained in the result paragraph.

Pretherapeutic tumor samples were collected from patients with locally advanced rectal cancer, who were treated at the Department of General, Visceral and Pediatric Surgery, University Medical Center, Göttingen, Germany. For the genome-wide screening, 45 patients were enrolled. These patients were identified in the database of the CAO/ARO/AIO-94 [18,19,20] and CAO/ARO/AIO-04 [21,22] trial of the German Rectal Cancer Study Group, and selected based on the criteria: locally advanced tumor, preoperative CRT, R0 resection, follow-up of at least 150 days, and availability of a pretherapeutic Formalin-Fixed Paraffin-Embedded biopsy (FFPE) showing invasive cancer. These patients were then split into two comparable groups based on the occurrence of distant metastases. For validation, 147 patients from the prospective collection were identified. Inclusion criteria were comparable to the screening set, but here the biomaterial used was preserved in RNAlater (in contrast to the biomaterial used for microarray analysis in the first set, which was FFPE material). Patients that were already analyzed in the screening set were excluded from validation analysis. Figure 4 illustrates the study design.

According to the CAO/ARO/AIO-94 [18,19,20] and CAO/ARO/AIO-04 [21,22] trial of the German Rectal Cancer Study Group, they received a preoperative CRT with a total radiation dose of 50.4 Gy (single dose of 1.8 Gy) accompanied by either an intravenous (iv) application of 5-FU or a combination of an iv-infusion of oxaliplatin and 5-FU according to the study protocol. Four to six weeks after preoperative CRT, surgery with quality controlled nerve-sparing TME was performed. Tumor regression grade (TRG) and posttherapeutic nodal stage (ypN) were assessed by experienced pathologists at the University Medical Center Göttingen, Germany. Written informed consent was obtained from all patients according to the guidelines approved by the local ethic committee respecting the principles of the Declaration of Helsinki. The clinical data are summarized in Supplementary Table S1. The follow-up period accounts for minimum 158 days, maximum 4111 days and mean 1620 days.

### 4.2. Tumor Biopsies, RNA Isolation and Microarray Analysis

Tumor biopsies, taken during the index rectoscopy, were immediately buffered formalin for the first set (*n* = 45); those for the validation set (*n* = 147) were transferred into RNAlater (Qiagen, Hilden, Germany). Tissue in RNAlater was stored over night at 4 °C to allow saturation of the entire biopsy. The following day, they were stored at −20 °C for long-term storage. Biopsies for FFPE tissue were stored overnight in 4% formalin and were processed the next day. RNA isolation from FFPE samples was carried out using the Qiagen AllPrep^®^ DNA/RNA FFPE kit (Qiagen, Hilden, Germany) according to the manufactures protocol after microdissection of the tumor tissue as previously described. RNA extraction from RNAlater biopsies was performed using TRIZOL (cat. no. 15596-018, Life Technologies, Rockeville, MD, USA) as previously described [12]. For these samples nucleic acid quantity, quality, and purity was determined using a spectrophotometer (NanoDrop ND1000, Thermo Fisher Scientific Inc., Waltham, MA, USA) and a 2100 Bioanalyzer (Agilent Technologies, Santa Clara, CA, USA). Expression profiling for miRNAs was carried out on the 60K Human microRNA Microarray (Agilent Technologies) as previously performed [82].

### 4.3. Semi-Quantitative Real-Time PCR

In an independent validation set of 147 patients, tumor samples were collected prospectively and processed. RNA was isolated as described above. Using the miScript Reverse Transcription Kit (Qiagen, Hilden, Germany) cDNA was generated from total RNA. Forward primers for miR-133b, miR-146b, miR-198, miR-223, miR-224, miR-23b, miR-3133, miR-320a, miR-34b, miR-3941, miR-4263, miR-450b-3p, miR-497, miR-515-5p, miR-518f, miR-573, miR-579, miR-612, and miR-802 were obtained from Qiagen’s database. For reverse primer, we used the miScript Universal Primer (Qiagen). Semi-quantitative real-time PCR was performed using the CFX 384™-Real-Time System (BIO-RAD, Hercules, CA, USA). The Quantification was carried out using QuantiTect SBYR Green PCR Master Mix (Qiagen, Hilden, Germany). Probes were analyzed in triplicates and assays were performed according to the manufacturer’s instruction.

### 4.4. Statistical Analysis

The miRNA array expression levels were analyzed using log2 transformation and quantile normalization [83]. Except for control spots, all 807 features were used without any a priori filtering. The data were analyzed with regard to clinical parameters overall survival (OS), disease-free survival (DFS), distant-metastasis-free survival (DMS), cancer-specific survival (CSS), TRG, and post-therapeutic nodal stage (ypN). For 14 miRNAs, we could find a significant association to at least two of four survival parameter (OS, DFS, CCS or DMS) with *p* < 0.05.

The miRNA PCR values of 19 miRNAs of 147 patients were normalized using housekeepers miR-202, miR-874 and small nuclear RNA U44 (SNORD44). These miRNAs were selected from the genome-wide screening and showed the least level of variability over all samples and represented miRNAs of high, medium, and low expression. Survival analysis of this data was performed as well as analyses of variance in order to assess two linear models of miRNA expression levels and clinical parameters [84]. These linear models included the expression level as dependent variable and the independent variables nodal state and tumor regression grade respectively. A significant outcome with *p* < 0.05 suggests an association of miRNA expression and the respective clinical data.

Survival data were visualized using Kaplan–Meier plots and the effect of the individual miRNAs on survival was assessed using Cox proportional hazards regression [85]. A Cox regression model was calculated miRNA-wise, correlating miRNA expression levels and time to event data. The overall survival was calculated as time from surgery until death. Cancer-specific survival time was measured from the date of resection to the date of death due to rectal cancer. Disease-free survival was calculated as the time from surgery to local recurrence or distant metastasis. Finally, the distant metastasis–free survival is the period until metastasis is detected. For visualization on Kaplan-Meier plots, patients were grouped depending whether they had an expression level above or below median expression level for a particular miRNA.

All analyses were performed using the free statistical software R (version 3.1, R Core Team, Wien, Austria) [86]. Linear models were computed using the “limma” package. Survival analysis was conducted using the R package “survival”. *p*-values < 0.05 were considered significant. In order to not exceed a false discovery rate of 5%, *p*-values were adjusted for multiple testing using the Benjamini–Hochberg method [87].

## Figures and Tables

**Table 1:** *p*-values of the 19 microRNAs concerning correlation to the clinical parameters.

MicroRNA	Overall Survival	Disease-Free Survival	Cancer-Specific Survival	Distant-Metastasis-Free Survival	ypN ^1^	TRG ^2^
miR-133b	0.1981	0.1797	0.1054	*0.032* *	0.8869	0.5895
miR-146b	0.5912	0.4065	0.7999	0.4282	*0.0465* *	0.9552
miR-198	0.5148	0.8378	0.4601	0.7771	0.3038	0.8873
miR-223	0.8491	0.8887	0.2834	0.9801	0.1327	0.8693
miR-224 *	0.1097	0.8256	0.2389	0.664	0.4927	0.736
miR-23c	0.6434	0.8418	0.445	0.8975	0.2371	0.8907
miR-3133	0.7581	0.6015	0.5951	0.4516	0.146	0.2699
miR-320a	0.2016	0.4967	0.3011	0.2985	0.2024	0.8778
miR-34b	0.9669	0.7904	0.9092	0.5506	0.6819	0.4109
miR-3941	0.0637	0.5656	0.0518	0.9055	0.7289	0.2858
miR-4263	0.3752	0.8541	0.3079	0.3768	0.9154	0.5357
miR-450b-3p	0.321	0.9334	0.3794	0.8234	0.4722	0.0878
miR-497	0.2883	0.8704	0.4852	0.6942	0.8824	0.4389
miR-515-5p	*0.0021* *	0.6666	*0.0132* *	0.9754	0.4235	0.867
miR-518f *	0.3482	0.8358	0.5293	0.5179	0.1116	0.0849
miR-573	*0.0364* *	0.8672	*0.0223* *	0.4698	0.0937	*0.0416* *
miR-579	*0.0092* *	0.695	*0.0186* *	0.896	0.0984	0.1266
miR-612	0.12	0.6907	0.0734	0.979	0.5931	0.4797
miR-802	*0.0231* *	0.1745	*0.0168* *	0.411	0.152	0.1402

^1^ ypN = pathological nodal stage post preoperative chemoradiotherapy; ^2^ TRG = tumor regression grade according to Dworak; * = *p* < 0.05. *Italic*: miRNAs with significant correlation to patients’ survival in microarray analysis, which were analyzed here in an independent set of 147 samples via quantitative real-time polymerase chain reaction (qPCR) for validation.

## Supplementary Materials

Supplementary materials can be found at http://www.mdpi.com/1422-0067/17/4/568/s1.

## Abbreviations

CRT	chemoradiotherapy
TME	total mesorectal excision
TRG	tumor regression grade
miRNA	microRNA
OS	overall-survival
CSS	cancer-specific-survival
LR	local recurrence
DM	distant metastasis
DMS	distant-metastasis-free survival
DF	disease-free-survival
ypN	post-chemoradiotherapy nodal status
f	female
m	male
