# Supplementary material for: Prognostic Value of MicroRNAs in Preoperative Treated Rectal Cancer
Source: Int J Mol Sci. 2016 Apr 15;17(4):568. doi: 10.3390/ijms17040568 (PMC4849024; doi:10.3390/ijms17040568)
Supplement: Supplementary file 1 [file ijms-17-00568-s001.pdf]

Supplementary Materials: Prognostic Value of microRNAs in Preoperative Treated Rectal Cancer

Azadeh Azizian, Ingo Epping, Frank Kramer, Peter Jo, Markus Bernhardt, Julia Kitz, Gabriela Salinas, Hendrik A. Wolff, Marian Grade, Tim Beißbarth, B. Michael Ghadimi and Jochen Gaedcke

Table S1. (**A**) Clinic parameters of 45 patients enrolled in this study for microarray analysis (set 1); (**B**) Clinic parameters of all 147 patients enrolled in this study for qPCR analysis (set 2).

Patient	Gender	Age	UICC	CSS Time (event)	LR Time (event)	DMS Time (event)	DFS Time (event)
1_1	m	56	III	125	125	125	125
1_2	m	59	II	21(1)	21	5(1)	5(1)
1_3	f	41	III	134	134	134	134
1_4	m	72	II	94	94	94	94
1_5	m	64	III	86	86	86	86
1_6	f	59	II	88	88	88	88
1_7	m	66	III	100	100	100	100
1_8	m	57	II	71(1)	32(1)	71(1)	32(1)
1_9	f	48	III	89	89	89	89
1_10	f	71	II	85	85	85	85
1_11	m	53	III	87	87	63(1)	63(1)
1_12	m	70	III	46	46	46	46
1_13	f	54	III	80	80	80	80
1_14	m	54	II	84(1)	59(1)	59(1)	59(1)
1_15	m	60	III	60	60	60	60
1_16	m	68	II	61	61	61	61
1_17	m	66	III	51(1)	51	25(1)	25(1)
1_18	f	81	III	61	61	61	61
1_19	m	66	III	59	59	59	59
1_20	m	59	III	61	61	61	61
1_21	m	81	II	39	39	39	39
1_22	m	62	II	34	34	34	34
1_23	f	64	III	89	89	89	89
1_24	f	67	II	80	80	80	80
1_25	f	71	III	83	73(1)	73(1)	73(1)
1_26	m	77	III	78	78	78	78
1_27	f	76	II	75	75	75	75
1_28	f	58	III	73	73	73	73
1_29	m	77	II	80	80	80	80
1_30	f	63	III	66	66	34(1)	34(1)
1_31	m	51	II	77	77	77	77
1_32	m	63	III	65	65	65	65
1_33	f	76	II	72	72	72	72
1_34	f	42	III	79	79	79	79
1_35	m	58	II	60	60	60	60
1_36	m	66	III	61	61	61	61
1_37	f	59	III	63	63	63	63
1_38	m	54	III	33(1)	33	27(1)	27(1)

Table S1. *Cont.*

Patient	Gender	Age	UICC	CSS Time (event)	LR Time (event)	DMS Time (event)	DFS Time (event)
1_39	m	57	III	60	60	60	60
1_40	m	61	III	58	58	58	58
1_41	f	64	II	73	47(1)	49(1)	47(1)
1_42	m	55	III	25(1)	25	4(1)	4(1)
1_43	m	50	II	69	69	13(1)	13(1)
1_44	m	36	III	67	67	29(1)	29(1)
1_45	m	78	II	60	60	60	60

(**A**)

Patient	Gender	Age	UICC	CSS Time (event)	LR Time (event)	DMS Time (event)	DFS Time (event)
1	m	67	II	46	46	46	46
2	m	71	II	29	29	29	29
3	m	46	II	50	50	50	50
4	f	56	IV	42	42	−4(1)	−4(1)
5	m	66	III	40	40	40	40
6	m	62	III	33	33	33	33
7	f	52	III	32	32	32	32
8	m	59	II	34	34	34	34
9	m	71	II	33	33	33	33
10	m	61	II	30	30	30	30
11	m	55	II	28	28	28	28
12	m	53	III	28	28	28	28
13	m	68	III	23	23	23	23
14	m	59	IV	26	26	7(1)	7(1)
15	m	70	III	13	13	13	13
16	m	64	III	17	17	17	17
17	f	78	II	35	35	35	35
18	f	68	IV	18	18	−3(1)	−3(1)
19	m	75	IV	10(1)	10	−3(1)	−3(1)
20	m	38	III	8	8	8	8
21	f	61	IV	6(1)	6	−1(1)	−1(1)
22	m	69	II	23	23	23	23
23	m	61	III	24	24	15(1)	15(1)
24	m	47	III	23	23	23	23
25	m	63	II	22	22	22	22
26	m	73	II	6	6	0(1)	0(1)
27	f	47	IV	13	13	−3(1)	−3(1)
28	f	73	II	16	16	16	16
29	m	75	II	13	13	13	13
30	m	52	III	12	12	12	12
31	m	61	III	13	13	13	13
32	f	49	III	13	13	13	13
33	m	70	III B	8	8	8(1)	8(1)
34	m	72	III	23	23	23(1)	23(1)
35	m	74	II	29	29(1)	29	29
36	m	65	II	5	5	5	5

Table S1. *Cont.*

Patient	Gender	Age	UICC	CSS Time (event)	LR Time (event)	DMS Time (event)	DFS Time (event)
37	m	62	II	124	124	124	124
38	m	60	II	111	111	111	111
39	m	68	III	135	135	135	135
40	f	71	III	120	120	120	120
41	m	65	III	91	91	91	91
42	m	49	II	109	109	109	109
43	m	53	II A	121	121	121	121
44	m	55	II	135	135	135	135
45	m	58	III	68(1)	42(1)	42(1)	42(1)
46	m	54	III	119	119	119	119
47	m	56	III	97	97	97	97
48	m	79	III	93(1)	93	50(1)	50(1)
49	f	61	III	62(1)	37(1)	42(1)	37(1)
50	f	60	III	107	107	107	107
51	m	62	II	86(1)	86	12(1)	12(1)
52	m	68	II	103	103	103	103
53	m	58	II	102	102	102	102
54	m	63	III	108	108	16(1)	16(1)
55	f	69	II	95	95	95	95
56	m	60	II	74(1)	40(1)	39(1)	39(1)
57	f	69	III	98	98	98	98
58	m	71	III	90	90	90	90
59	m	60	III	79	79	48(1)	48(1)
60	m	65	III	94	94	94	94
61	m	71	II	79	79	79	79
62	f	67	II	84	84	84	84
63	f	73	III	64	64	64	64
64	m	80	III	59	59	59	59
65	m	68	III	60	60	60	60
66	m	70	III	40(1)	40	11(1)	11(1)
67	m	66	III	5(1)	5	0(1)	0(1)
68	m	57	III	60	60	60	60
69	m	61	II	89	89	89	89
70	m	71	III	79	79	79	79
71	m	67	III	79	79	79	79
72	m	47	III	73	73	73	73
73	m	55	III	78	78	78	78
74	m	49	II	75	75	75	75
75	m	78	III	63	63	63	63
76	f	68	III	46(1)	46(1)	46	46(1)
77	m	35	III	75	75	75	75
78	m	71	III	15(1)	15	9(1)	9(1)
79	m	60	II	68	68	68	68
80	f	66	III	7(1)	7	6(1)	6(1)
81	m	43	III	21(1)	21	−4(1)	−4(1)
82	f	48	III	60	60	60	60
83	m	47	III	60	60	60	60

Table S1. *Cont.*

Patient	Gender	Age	UICC	CSS Time (event)	LR Time (event)	DMS Time (event)	DFS Time (event)
84	m	54	II	62	45(1)	0(1)	0(1)
85	f	74	IV	32(1)	32	0(1)	0(1)
86	m	80	II	56(1)	42(1)	56	42(1)
87	m	65	III	60	60	60	60
88	f	75	III	24(1)	24	19(1)	19(1)
89	m	63	III	50	50	50	50
90	m	76	III	51	51	51	51
91	m	69	III	60	60	60	60
92	m	73	III	61	61	61	61
93	m	73	III	59	59	59	59
94	m	55	III	54	33(1)	34(1)	33(1)
95	f	63	III	29(1)	29	14(1)	14(1)
96	f	36	III	14(1)	14	8(1)	8(1)
97	m	64	III	23	23	23	23
98	m	59	III	48(1)	48	48	48
99	m	75	III	20	20	13(1)	13(1)
100	f	51	III	36	18(1)	36	18(1)
101	f	46	III	48	48	48	48
102	m	54	II	49	49	49	49
103	m	61	III	52	52	52	52
104	f	59	III	38	38	38	38
105	f	71	III	46(1)	46	33(1)	33(1)
106	m	60	III	48	48	48	48
107	m	81	III	36	36	36	36
108	f	73	III	47	47	47	47
109	m	71	II	48	48	48	48
110	m	64	III	50(1)	25(1)	46(1)	25(1)
111	m	64	II	48	48	48	48
112	m	72	IV	44	44	−4(1)	−4(1)
113	f	70	III	48	48	48	48
114	f	65	III	48	48	48	48
115	m	55	III	48	48	5(1)	5(1)
116	f	56	III	38	38	38	38
117	m	66	III	41	41	41	41
118	f	47	II	52(1)	52	11(1)	11(1)
119	m	80	III	48	48	48	48
120	m	67	III	42	42	42	42
121	m	55	II	36	36	36	36
122	f	74	III	48	48	26(1)	26(1)
123	m	62	III	42	42	42	42
124	f	50	III	36	36	36	36
125	f	77	III	47	47	47	47
126	f	42	III	36	36	36	36
127	m	73	III	34	34	34	34
128	m	42	IV	42	42	0(1)	0(1)
129	m	62	III	38	38	38	38
130	m	42	II	35	35	35	35

Table S1. *Cont.*

Patient	Gender	Age	UICC	CSS Time (event)	LR Time (event)	DMS Time (event)	DFS Time (event)
131	m	68	IV	33	33	−3(1)	−3(1)
132	f	79	III	26	26	26	26
133	f	64	III	38	38	38	38
134	m	61	III	37	37	27(1)	27(1)
135	m	72	III	33	33	19(1)	19(1)
136	m	63	III	24	24	24	24
137	f	52	III	21	21	21	21
138	m	67	II	24	24	24	24
139	f	69	IV	24	24	−4(1)	−4(1)
140	m	70	II A	21	21	21	21
141	f	64	III	24	24	24	24
142	f	53	II	24	24	24	24
143	m	62	II A	21	21	21	21
144	m	58	III B	20	20	20	20
145	m	63	II A	16	16	16	16
146	m	76	III B	20	20	20	20
147	m	71	III B	17	17	17	17

(**B**)

f = female, m = male, CSS time = cancer specific survival time (in months), LR time = time without a local recurrence of the tumor (in months), DMS time = time without a distant metastasis (in months), DFS time = time of disease free survival (in months), (1) = event.

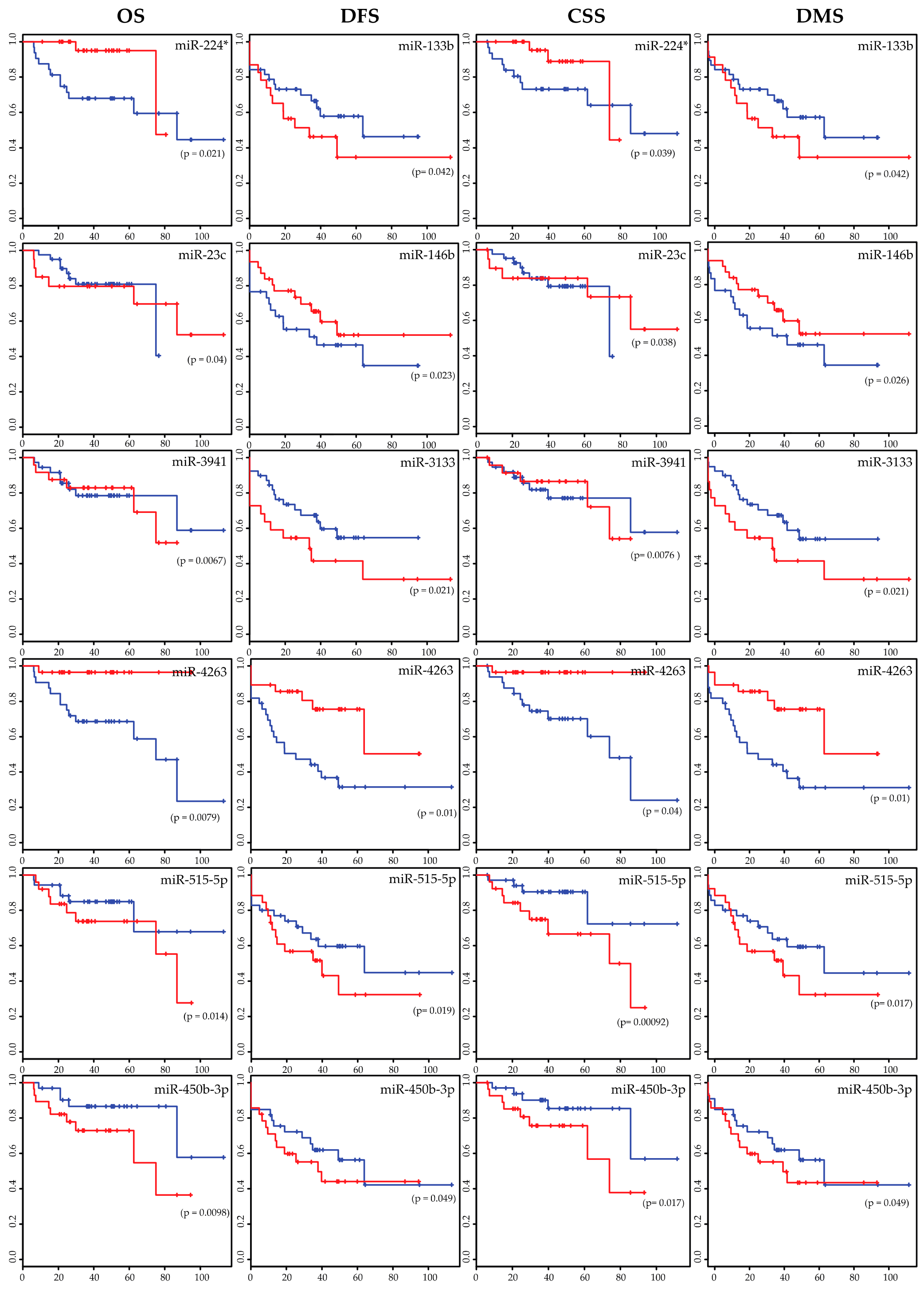

Figure S1. *Cont.*

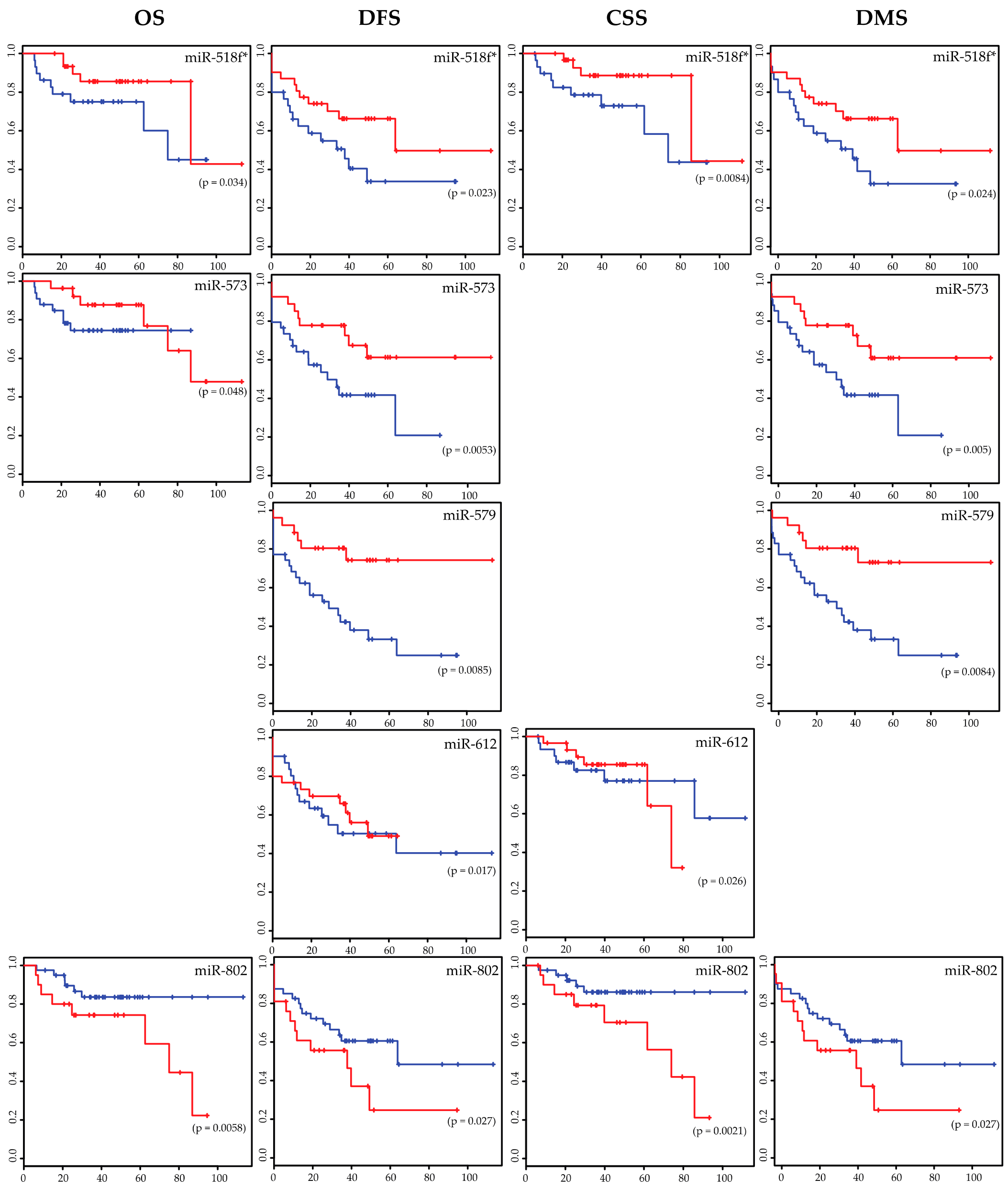

Figure S1. Survival plots showing significant correlation to microRNA expression determined by microarray analysis. Red curves represent patients with a high expression of the microRNA, blue curves represent patients with a low expression of the microRNA. Curves showing no significant correlations are not shown. OS, overall survival; DFS, disease-free survival; CSS, cancer-specific survival; DMS, distant-metastasis-free survival.
